# Evaluation of the rotary kinematics between actual and set speeds of X-Smart Plus, VDW.Silver and iRoot motors

**DOI:** 10.1590/0103-6440202304953

**Published:** 2023-05-15

**Authors:** Renata de Castro Monteiro-Netto, Dieimes Braambati, Rodrigo Arruda-Vasconcelos, Adriana de-Jesus Soares, Marcos Frozoni

**Affiliations:** 1 Department of Restorative Dentistry, Division of Endodontics, São Leopoldo Mandic Dental School, Campinas, São Paulo, Brazil.; 2 Department of Restorative Dentistry, Division of Endodontics, Piracicaba Dental School, State University of Campinas - UNICAMP, Piracicaba, SP, Brazil.

**Keywords:** Endodontics, kinematics, rotary motion

## Abstract

The present study evaluated the actual rotational speed of three different endodontic motors compared to the values provided by the manufacturers. A total of three endodontic motors (X-Smart Plus, VDW.Silver, and iRoot) were tested at 400 rpm and 800 rpm and 2 N/cm^2^ torque. The kinematics of the devices was recorded by using a custom angle-measuring disc with a 50-mm diameter attached to the handpiece provided by the manufacturer, whereas their movement was captured by a high-speed camera at 2,400 frames *per* second, 800 x 800 pixel-resolution and distance of 0.3 m from the target object. Statistical analysis was performed at a significance level of 5%. At 400 rpm, the iRoot motor had a value of 17.94 rpm above that indicated by the manufacturer, which was significantly different from those of X-Smart Plus (5.20 rpm below that indicated by the manufacturer) and VDW.Silver (0.62 rpm above that indicated by the manufacturer) motors (*P* < 0.05). At 800 rpm, the iRoot motor had a value of 51.34 rpm below that indicated by the manufacturer, whereas the X-Smart Plus motor had a value of 13.00 rpm below that indicated by the manufacturer (*P* > 0.05). The VDW.Silver motor statistically differed from the iRoot and X-Smart Plus ones, showing a value of 1.68 rpm above that indicated by the manufacturer. In conclusion, the X-Smart Plus, VDW.Silver, and iRoot motors showed lower variations in the rotational speed values compared to those reported by their manufacturers. The endodontic motors presented different behaviors between them, with the VDW.Silver motor presents the most accurate values and the iRoot presents the most divergent values.

## Resumo

O presente estudo avaliou a real velocidade rotacional de três diferentes motores endodônticos comparados aos valores fornecidos pelos fabricantes. Um total de três motores endodônticos (X-Smart Plus, VDW.Silver e iRoot) foram testados a 400 rpm e 800 rpm e torque de 2 N/cm^2^. A cinemática dos dispositivos foi verificada utilizando-se um disco customizado de 50 mm de diâmetro acoplado ao contra ângulo fornecido pelo fabricante e o movimento foi capturado através de uma câmera ajustada em 2400 quadros por segundo com resolução de 800 x 800 pixels distante 0,3 m do objeto alvo. A análise estatística foi realizada com nível de significância de 5%. A 400 rpm, o motor iRoot apresentou 17,94 rpm acima do indicado pelo fabricante, sendo o valor significativamente diferente do X-Smart Plus (5,20 rpm abaixo do indicado pelo fabricante) e VDW.Silver (0.62 rpm acima do indicado pelo fabricante) (*P* > 0,05). A 800 rpm, iRoot apresentou 51.34 rpm abaixo do indicado pelo fabricante e X-Smart Plus 13,00 rpm abaixo do indicado pelo fabricante (*P* > 0,05). VDW.Silver foi estatisticamente diferente de iRoot e X-Smart Plus, apresentando 1,68 rpm acima do indicado pelo fabricante. Concluiu-se que os motores X-Smart Plus, VDW.Silver e iRoot apresentaram baixa variação na velocidade rotacional quando comparada ao fornecido pelo fabricante. Os motores endodônticos apresentaram diferentes comportamentos entre eles, sendo que o motor VDW.Silver apresentou valores mais acurados enquanto o iRoot foi o mais divergente.

## Introduction

The main objective of root canal treatment is to prevent or eliminate apical periodontitis ^1^. In this context, mechanical preparation is recognized as a key step for eliminating/reducing bacteria and their by-products from the root canal system ^1^ ^,^ ^2^ ^,^ ^3^ ^,^ ^4^,^5^. Studies have reported that bacterial reduction ranges from 80% to over 99% ^6^ ^,^ ^7^ ^,^ ^8^ ^,^ ^9^.

Over the past years, technological improvements have been proposed in endodontics (e.g., nickel-titanium alloy, instruments with different taper designs, surface finishing, kinematics) aiming to increase safety in clinical procedures, especially those involving narrow curved root canals, achieve faster treatment, improve maintenance of the original canal anatomy, and produce predictable outcomes compared to conventional techniques for root canal treatment ^10^ ^,^ ^11^ ^,^ ^12^.

Overall, cyclic and torsional fatigue occurs due to the stress of tension and compression at the point of maximum flexure, especially in curved root canals ^13^ ^,^ ^14^ with the latter occurring when part of the instrument binds to the root canal while the shank continues to rotate ^15^. Several factors can affect the resistance of the instrument, such as its dimensions, tip size and tapper, cross-section design, the chemical composition of the metallic alloy, thermo-mechanical processes applied during manufacturing, remaining defects after the machining process, and experience of the operator ^12^ ^,^ ^16^. Furthermore, variation in rotational speed is an important factor regarding clinical performance and fracture resistance of the endodontic instruments ^17^. It has previously been reported that instrument rotation at higher speeds is more likely to fracture than at low speeds ^18^. 

Numerous studies have extensively evaluated the use of different instruments regarding design, metallurgy, mechanical performance, disinfection capacity, shaping ability, dentinal microcrack formation, and post-operative pain ^1^ ^,^ ^12^ ^,^ ^19^ ^,^ ^20^ ^,^ ^21^ ^,^ ^22^. On the other hand, the literature is limited to studies investigating the rotational speed in different endodontic electric motors. Increased rotational speed applied to nickel-titanium rotary instruments may result in its fracture due to the higher number of cycles applied to the instruments within the same period ^23^. Thus, the aim of this study was to evaluate the actual rotational speed of three different endodontic motors by comparing the values provided by their manufacturers. The null hypothesis tested was that there would be no difference between actual and set speeds of X-Smart Plus, VDW.Silver, and iRoot motors.

## Material and methods

### Endodontic motors and its kinematics’ evaluation

Three endodontic electric motors with their handpieces were selected after calibration as recommended by the manufacturers.

The endodontic motors used in this study were X-Smart Plus (Dentsply Sirona, Ballaigues, Switzerland), VDW.Silver (VDW GmbH, Munich, Germany), and iRoot (Easy Equipamentos Odontológicos, Belo Horizonte, MG, Brazil). All endodontic motors were tested at set speeds of 400 and 800 rpm, both with a torque of 2 N/cm^2^.

The evaluation of the kinematics was adapted from previous studies ^24^ ^,^ ^25^ ^,^ ^26^.

**Figure 1:**
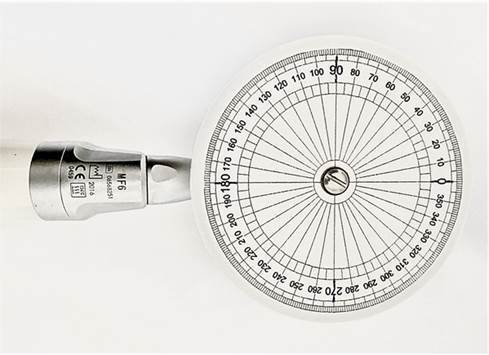
Custom angle-measuring disc with a 50-mm diameter used as a target object.

Briefly, a custom angle-measuring disc with a 50-mm diameter (Figure 1) and a shaft designed to fit into the chuck of the handpiece was manufactured from polypropylene by a computer numerical control machine. Four major lines at every 90° and 68 minor lines at every 5° were engraved on the disc as reference lines for visual kinematic analysis. One of the major lines was marked with black ink to serve as the main reference for image analysis. The target object (*i.e.,* measuring disc) was inserted into the contra-angle of each endodontic motor (X-Smart Plus, VDW.Silver or iRoot), which was then attached to a clamp in front of a lens (M31711) coupled to a high-speed camera (Phantom VEO 710, Infinity Photo Optical Company, Centennial, CO, USA) and kept at a distance of 0.3 m. All parts and surfaces were aligned by using a water level. The camera was set to high-speed video mode at 2400 frames *per* second (fps) with 800 x 800-pixel resolution and recorded in CINE format. The recording environment was illuminated by using a light source (GS Vitec SN: 6500068, GS Vitec Gmbh, Bad Soden-Salmünster, Germany) with an output of 7000 lumens. Each endodontic motor was recorded 4 times for 10 seconds.

### RPM calculation

**Figure 2:**
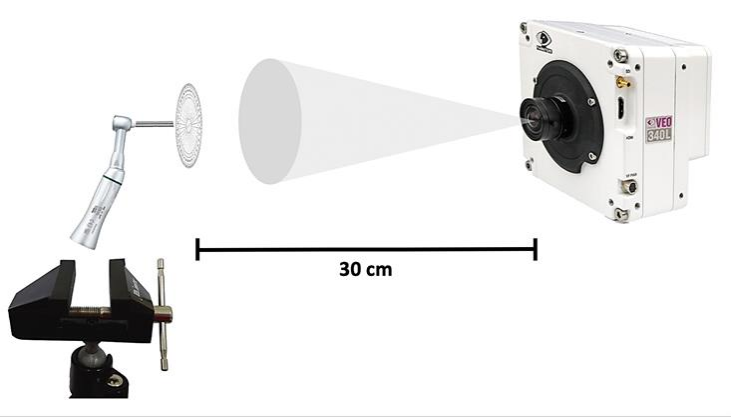
Apparatus for image acquisition - a set consisting of a camera, lenses, the target object and handpiece.

A total of 10 complete cycles of rotary movements were randomly selected and analyzed during the 10-second period of recording, thus rendering the accurate time of each cycle of movements. Next, image processing and analysis software (Vision Research®, Inc. Headquarters, Wayne, NJ, USA) was used to assess the time required to complete 1 cycle of rotary motion (Figure 2). This calculation included 10 selected cycles and the results were converted into microseconds in order to obtain the number of revolutions *per* minute (rpm). Each endodontic motor was recorded 4 times for 10 seconds.

#### Statistical analysis

Statistical analysis was performed by using SPSS software (Chicago, IL, USA). Based on a previous study ^24^ showing an intraclass correlation coefficient (ICC) of 0.1139, sample size calculation for α = 0.05, and power (1-β) = 0.8, it was necessary to have four recordings of each of the six groups to obtain a minimum ICC value of 0.75. Shapiro-Wilk test was used to verify the data normality, whereas non-parametric Kruskal-Wallis and Dunn tests were used for comparisons between the three endodontic motors at both rotations. The significance level was set to 5%.

## Results

The values of ICC for X-Smart Plus, VDW.Silver and iRoot motors were 0.999, 1.0, and 0.987, respectively.

At 400 rpm and 2N/cm^2^, the iRoot motor showed a mean rotational speed of 417.94 rpm (17.94 rpm above that indicated by the manufacturer), which was significantly different from those of X-Smart Plus (394.80 rpm; 5.20 rpm below that indicated by the manufacturer) and VDW.Silver (400.62 rpm; 0.62 rpm above that indicated by the manufacturer) motors (*P* < 0.05). No statistically significant differences were observed between the X-Smart Plus and VDW.Silver motors (*P* > 0.05) (Table 1).

At 800 rpm and 2 N/cm^2^, the iRoot motor showed a mean rotational speed of 748.64 rpm (51.34 rpm below that indicated by the manufacturer), with no statistically significant difference compared to the X-Smart Plus one (787.00 rpm; 13.00 rpm below that indicated by the manufacturer) (*P* > 0.05). Conversely, the VDW.Silver motor showed a mean rotational speed of 801.68 rpm (1.68 above that indicated by the manufacturer), which was statistically different from those of the iRoot and X-Smart Plus ones (*P* < 0.05) (Table 1).

**Table 1:** Mean (standard deviation) of revolutions per minute (rpm) and absolute error (rpm) in relation to the set rotational speed provided by the manufacturer.

Endodontic motor	400 rpm		800 rpm	
	Actual speed	Absolut error	Actual speed	Absolute error
X-Smart Plus	394,80 (0,11)	-5,20 (0,11) ^A^	787,00 (0,54)	-13,00 (0,54) ^AB^
VDW Silver	400,62 (0,35)	0,62 (0,35) ^AB^	801,68 (0,56)	1,68 (0,56) ^A^
iRoot	417,94 (0,98)	17,94 (0,98) ^B^	748,66 (3,32)	-51,34 (3,32) ^B^

Different uppercase letters in the same column denote statistically significant differences (*P* < 0.05).

## Discussion

With the advent of technology in the field of endodontics, numerous systems have been proposed for the mechanical preparation of root canals. Currently, a wide variety of instruments with different tip and taper sizes, cross-section designs, kinematics, and surface treatment are available to the clinician. Over the years, several studies have investigated the fracture resistance of endodontic instruments ^12^ ^,^ ^19^ ^,^ ^27^ ^,^ ^28^ ^,^ ^29^. However, to the best of the authors’ knowledge, the literature is limited to studies evaluating the actual rotational speed of different endodontic motors compared to the values provided by the manufacturers.

The method for assessing the kinematics of endodontic motors has been successfully employed ^24^ ^,^ ^25^. In the present study, the camera was set to high-speed video mode at 2400 fps with 800 x 800-pixel resolution, whereas previous studies have used 1000 fps and 224 x 64 pixel-resolution ^24^ as well as 1200 fps and 336 x 96-pixel resolution ^25^. The improvement in the quality of imaging acquisition can lead to a more precise determination of the kinematics of endodontic motors.

The current study proved to be relevant as it is intimately related to the safety for performing root canal treatment. According to Li et al. ^23^, a high rotational speed results in the fracture of the nickel-titanium endodontic instruments in a reduced period as a higher number of cycles are applied. Therefore, it is possible to infer that the three endodontic motors can present different behaviors regarding the lifespan of endodontic files during the root canal treatment.

Overall, the results showed that there was a low divergence in the rotational speed values provided by the manufacturers and those detected in the present study, which is corroborated by previous studies on reciprocating motion ^24^ ^,^ ^25^. On the other hand, it is important to highlight that statistical differences were observed between the different endodontic motors.

A potential explanation for the differences observed between the endodontic motors regarding their rotational speed is that both the X-Smart Plus and the VDW.Silver motors have a battery and a connection cable to their handpieces, while the iRoot motor is a wireless device. The ability of batteries to keep the engines working with stability and precision should be the subject of further studies.

A strength of this study relies on the intraclass correlation coefficient of the tested endodontic motors (X-Smart Plus: 0.999, VDW.Silver: 1.000, and iRoot: 0.987), demonstrating excellent reliability of the measurements. Notably, the quality of image acquisition was superior (2,400 fps; 800 x 800 pixels) than those reported by previous studies (1,200 fps; 360 x 360 pixels) ^25^ and (1,000 fps; 224 x 64 pixels) ^24^. On the other hand, a potential limitation of this study is that the endodontic motors were tested with no variation in workload or torque, which may influence the results if used in a clinical situation. Additionally, a single endodontic motor of each manufacturer (iRoot, X-Smart, and VDW.Silver) was used during the investigation. Importantly, only brand-new endodontic motors were used during the tests.

This study shed a light on the actual kinematics of different endodontic motors as all of them presented different speeds than those reported by their manufacturers. Importantly, further investigations should be performed to test other endodontic motors under different torques and workloads to better simulate procedures and aid the decision-making process in the clinical practice.

In conclusion, the X-Smart Plus, VDW.Silver and iRoot endodontic motors showed low variations in the rotational speed than those reported by the manufacturers. The endodontic motors presented different behaviors between them, being VDW.Silver the most accurate endodontic motor and the iRoot motor is the most divergent between them.
